# Smoothness of movement in idiopathic cervical dystonia

**DOI:** 10.1038/s41598-022-09149-1

**Published:** 2022-03-24

**Authors:** Antonio Caronni, Pietro Arcuri, Ilaria Carpinella, Alberto Marzegan, Tiziana Lencioni, Marina Ramella, Alessandro Crippa, Denise Anastasi, Marco Rabuffetti, Maurizio Ferrarin, Anna Castagna

**Affiliations:** IRCCS Fondazione Don Carlo Gnocchi, via Alfonso Capecelatro 66, 20148 Milan, Italy

**Keywords:** Dystonia, Dystonia, Motor control, Biomedical engineering, Physiology

## Abstract

Smoothness (i.e. non-intermittency) of movement is a clinically important property of the voluntary movement with accuracy and proper speed. Resting head position and head voluntary movements are impaired in cervical dystonia. The current work aims to evaluate if the smoothness of voluntary head rotations is reduced in this disease. Twenty-six cervical dystonia patients and 26 controls completed rightward and leftward head rotations. Patients’ movements were differentiated into “towards-dystonia” (rotation accentuated the torticollis) and “away-dystonia”. Smoothness was quantified by the angular jerk and arc length of the spectrum of angular speed (i.e. SPARC, arbitrary units). Movement amplitude (mean, 95% CI) on the horizontal plane was larger in controls (63.8°, 58.3°–69.2°) than patients when moving towards-dystonia (52.8°, 46.3°–59.4°; P = 0.006). Controls’ movements (49.4°/s, 41.9–56.9°/s) were faster than movements towards-dystonia (31.6°/s, 25.2–37.9°/s; P < 0.001) and away-dystonia (29.2°/s, 22.9–35.5°/s; P < 0.001). After taking into account the different amplitude and speed, SPARC-derived (but not jerk-derived) indices showed reduced smoothness in patients rotating away-dystonia (1.48, 1.35–1.61) compared to controls (1.88, 1.72–2.03; P < 0.001). Poor smoothness is a motor disturbance independent of movement amplitude and speed in cervical dystonia. Therefore, it should be assessed when evaluating this disease, its progression, and treatments.

## Introduction

Idiopathic cervical dystonia is a neurologic disease characterised by involuntary contractions of cervical muscles, which causes abnormal resting positions and movements of the head, neck and shoulders^1^. The severity of the motor impairment can be disabling^2^, negatively affecting the quality of life^3^.

The most common motor sign of cervical dystonia is a postural abnormality presented with the deviation of the head from its physiological neutral position in resting conditions^4^. However, phenomenology (i.e. motor signs) of cervical dystonia is more complex, including the impairment of voluntary motion and the presence of involuntary movements, such as spasms, tremors and jerks^4^.

Compared to healthy controls, head movements in people with cervical dystonia are characterised by reduced amplitude^5,6^ and velocity^5–7^, which are common features of the pathological movement in general^8^. In addition, pathological movement is frequently characterised by poor smoothness.

A movement appears smooth when it develops without interruptions and, the other way round, movements with intermittency look jerky^9^. Loss of smoothness is a major sign in ataxia, such as the cerebellar one^10–12^, but it is also present in many other neurological diseases, such as stroke^13,14^, multiple sclerosis^15,16^ and Parkinson’s disease^17–19^.

Notably, movement smoothness is routinely evaluated in the bedside neurological examination, further highlighting the importance of this feature during movement assessment. For example, movement’s interruptions and decomposition are sought in the diadochokinesia^10,20^, finger-to-nose and knee-tibia^11^ tests.

Movement smoothness (defined as continuousness, non-intermittency of movement^21^) is immediately apparent from the time course of movement velocity. Healthy and well-trained movements occurring from point to point along a single direction (e.g. planar reaching movements, head rotations, eye saccades) are characterised by a typical bell-shaped speed profile^22^ and high smoothness^9^. When movement intermittency is present, this bell-shaped profile is disrupted by dips (which flag the deceleration of movement followed by its acceleration), up to periods of zero speed (which indicate true movement interruptions)^9^.

Even if smoothness is a relatively intuitive concept^23^, its measurement poses some real challenges. Although there is no consensus on the best method to quantify smoothness, two measures seem to have the highest validity: the log dimension-less jerk (LDLJ) and the spectral arc length measure, commonly referred to as SPARC^9^. While LDLJ represents the mean rate of change of the squared acceleration of a movement, i.e. the mean squared magnitude of the third time derivative of position^22,24^, SPARC stems from the observation that high-frequency components are abundant in unsmooth movements. In contrast, smooth movements are composed mainly of low-frequency components^23^. For example, compared with a smooth movement, a jerky movement results in a Fourier spectrum of the speed profile with many peaks at high frequency. SPARC turns this finding into a measure by simply calculating the length of the speed spectrum^23^.

Importantly, smoothness measures are sensitive to movement amplitude and velocity^8^. A positive relationship has been described between smoothness and speed, with fast movements smoother than the slower ones. It has been even suggested that high smoothness actually promotes more rapid movements^25^.

This relationship between movement speed and smoothness is of the most significant importance when smoothness is compared in patients and controls, given that, as reported above, patients often show a reduction of both movement amplitude and speed. With this regard, it has been clearly proposed that movement amplitude and speed should be carefully controlled when smoothness is investigated^25^.

Intermittency of voluntary movements, such as voluntary head movements, has also been reported in cervical dystonia^7^. However, to our knowledge, a detailed investigation of the smoothness of movement in cervical dystonia is missing, as well as its relationship with basic movement parameters such as amplitude and speed.

The aim of the current work is twofold. First, as previously suggested, we will investigate if the amplitude and speed of head rotations in cervical dystonia are different from controls. Second, we will assess if, net of any difference in these two parameters, head rotations in cervical dystonia are also less smooth than in controls’.

## Results

### Sample description

Patients’ clinical characteristics are given in Table 1.

**Table 1:** Patients’ clinical characteristics.

ID	Gender^a^	Age (years)	Disease duration (years)	Phenotype^b^	Dystonic tremor^c^	TWSTRS severity	TWSTRS total
1	F	49	7	TC Lt	N	16	37.2
2	F	55	4	TC Lt	Y	21	54.75
3	M	64	10	TC Rt	N	14	35.5
4	M	69	14	TC Rt, LC Rt	N	20	32
5	F	54	15	TC Rt	Y	16	39
6	M	79	27	TC Rt, LC Lt	Y	18	29
7	F	48	7	TC Lt	Y	12	39
8	M	54	10	TC Lt, LC Rt	N	21	48.25
9	F	64	23	TC Lt	N	18	28.25
10	F	49	5	TC Lt, LC Lt	N	11	30.75
11	F	45	19	TC Lt	N	24	44
12	F	73	3	TC Lt, LC Lt	N	19	33.25
13	M	42	7	TC Lt, LC Lt	N	19	37
14	F	58	2	TC Lt	N	15	36.75
15	M	53	4	TC Rt, LC Lt	N	18	42.15
16	M	46	12	TC Lt, LC Rt	N	12	26.25
17	M	40	1	TC Rt	N	15	30
18	F	46	2	TC Lt	Y	21	45.5
19	M	37	2	TC Rt, LC Lt	N	20	51.25
20	M	53	2	TC Lt	N	9	16
21	F	53	13	TC Lt	N	11	26.5
22	F	43	2	TC Rt, LC Lt	N	20	35
23	F	68	22	TC Rt	Y	16	32.5
24	F	44	1	TC Lt	N	15	50
25	M	54	3	TC Rt	N	19	33.5
26	M	56	31	TC Rt	N	8	12.75
Summary^d^	F = 14 M = 12	53 (11.5)	7 (11.5)	TC = 26 TC + LC = 10	Y = 6 N = 20	17 (5.5)	35.25 (11.17)

^a^gender: *F* female, *M* male.

^b^Phenotype: *TC* torticollis, *LC* laterocollis, *Rt* right, *Lt* left.

^c^Dystonic tremor: presence (Y) or absence (N) of tremor in addition to dystonic posturing.

^d^Summary: counts and median (interquartile range) are given in the last row.

In accordance with the inclusions criteria, all patients had torticollis (15 to the left and 11 to the right). In addition to torticollis, ten patients also had some laterocollis (seven to the left and three to the right). In patients with torticollis and laterocollis, torticollis was the main clinical feature. Six patients also had dystonic tremor. Only one patient had torticollis, laterocollis and dystonic tremor.

The Toronto Western Spasmodic Torticollis Rating Scale (TWSTRS) severity score ranged from 8 to 24, with a median score (17) substantially corresponding to the middle of the scale. This finding suggests that, from a clinical perspective, the motor impairment of the patients’ sample was of moderate severity.

Controls’ age (median = 47, interquartile range = 21.75 years) and gender distribution (12 females) were comparable to the patients’ ones (53, 11.5 years; 14 females).

### Head rotation: amplitude and velocity

**Figure 1:**
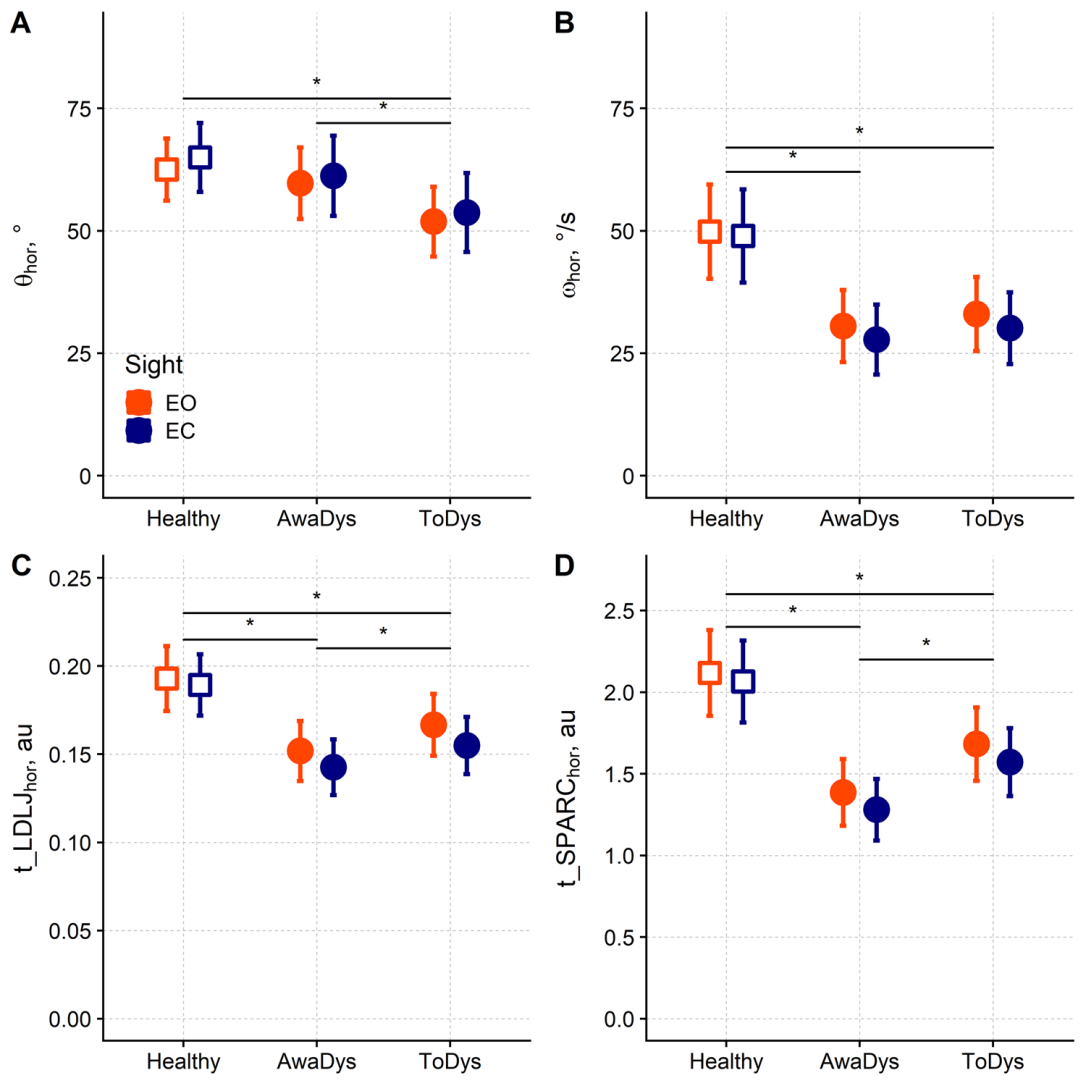
Amplitude, velocity and smoothness of head rotations in controls and patients with cervical dystonia. *Healthy* controls’ movement, *AwayDys* patients’ away dystonia movement, *ToDys* patients’ to dystonia movement, *EO* eyes open, *EC* eyes closed, *θ* movement amplitude, *ω* movement velocity, *t_LDLJ* transformed log-dimensionless jerk, *t_SPARC* transformed spectral arc length metric, *hor* horizontal plane, *au* arbitrary units. Mean and the 95% confidence interval are given. As customary, * marks a significant difference between two values (horizontal bar).

The results related to rotations in the horizontal plane (prime movements) are reported in Fig. 1A,B.

Rotation amplitude on the horizontal plane (θ_hor_) was significantly different among movement types (generalised linear mixed-effects models followed by the Type III Wald chi-square test: *χ*^2^_2_ = 20.49, *P* < 0.001). In particular, movement “towards-dystonia” (ToDys) was smaller in amplitude than movement “away-dystonia” (AwayDys) and the healthy movement (*P* ≤ 0.006) (Fig. 1A).

Horizontal rotation velocity (ω_hor_) was also significantly different (*χ*^2^_2_ = 22.53, *P* < 0.001) among movement types, with the healthy movement being faster than AwayDys and ToDys movements (*P* < 0.001) (Fig. 1B).

No difference was found either for θ_hor_ or for ω_hor_ between moving with the open and the closed eyes. The interaction between movement type and vision was also not significant.

The results related to the rotation amplitude in the coronal (θ_cor_) and sagittal planes (θ_sag_) (“spurious” movements) are shown in Table 2. θ_cor_ was larger in AwayDys movement compared to ToDys (*P* = 0.006) and healthy (*P* = 0.032) movements. No difference was found in the sagittal plane.

**Table 2:** Amplitude and velocity of spurious rotations of the head in the coronal and sagittal planes.

Rotation plane	Variable	Healthy Mean (95% CI)^a^	AwayDys Mean (95% CI)^a^	ToDys Mean (95% CI)^a^	Wald χ^2^ (P)^b^
Coronal	θ (°)	14.1 (11.6–20.8)	18.1*^†^ (15.4–20.8)	13.5 (11.1–15.9)	10.40 (0.006)
	ω (°/s)	31.8 (29.2–34.4)	27.3* (24.9–29.8)	28.8 (26.4–31.3)	8.74 (0.013)
Sagittal	θ (°)	9.16 (7.36–11.0)	9.32 (7.54–11.1)	8.64 (6.90–10.4)	0.86 (0.652)
	ω (°/s)	26.8 (24.8–28.8)	22.8* (20.9–24.6)	23.6* (21.7–25.4)	8.79 (0.012)

*Statistically significant differences with respect to Healthy (post-hoc test, P corrected according to Bonferroni).

^†^Statistically significant differences with respect to ToDys (post-hoc test, P corrected according to Bonferroni).

^a^Movement type: Healthy, controls’ movement; AwayDys, patients’ movement in the “away dystonia” direction; ToDys, patients’ movement in the “towards dystonia” direction; *95% CI *95% confidence interval.

^b^Statistics: *Wald χ*^*2*^, type III Wald chi-square statistics (two degrees of freedom); *P*, type 1 error probability of the Wald chi-square test.

Rotation velocity in the coronal plane (ω_cor_) was higher in healthy compared to AwayDys movement (*P* = 0.007). Rotation velocity in the sagittal plane (ω_sag_) was higher in the healthy condition compared to AwayDys and ToDys movements (*P* ≤ 0.015) (Table 2).

No significant difference between visual conditions and no significant interactions between movement type and vision were found for any variable.

### Head rotation: movement smoothness

Two indices were used to quantify the smoothness of movement in the horizontal plane: the log dimension-less jerk (LDLJ_hor_) and the Spectral Arc length (SPARC_hor_). Both of them were transformed (t_) so that higher values indicate higher smoothness.

t_LDLJ_hor_ varied according to the movement type (*χ*^2^_2_ = 29.77, *P* < 0.001) (Fig. 1C). In patients, t_LDLJ_hor_ was lower in AwayDys than in ToDys (*P* < 0.001). t_LDLJ_hor_ was larger in healthy movements than both AwayDys and ToDys movements (*P* ≤ 0.001).

Regardless the movement type, t_LDLJ_hor_ was larger with the open eyes (0.171, 95% confidence interval, 95% CI 0.160–0.181) than with the closed eyes (0.162, 95% CI 0.153–0.172; *χ*^2^_1_ = 4.26, *P* = 0.039), while the interaction between movement type and vision was not significant.

t_SPARC_hor_ also changed according to the movement type (*χ*^2^_2_ = 51.54, *P* < 0.001; Fig. 1D). In agreement with t_LDLJ_hor_, t_SPARC_hor_ was also smaller in AwayDys than ToDys (*P* < 0.001) and larger in healthy movements compared to AwayDys and ToDys (*P* < 0.001). Neither vision nor interaction between movement type and vision did affect t_SPARC_hor_.

### Head rotation: effect of movement amplitude and speed on the smoothness

**Figure 2:**
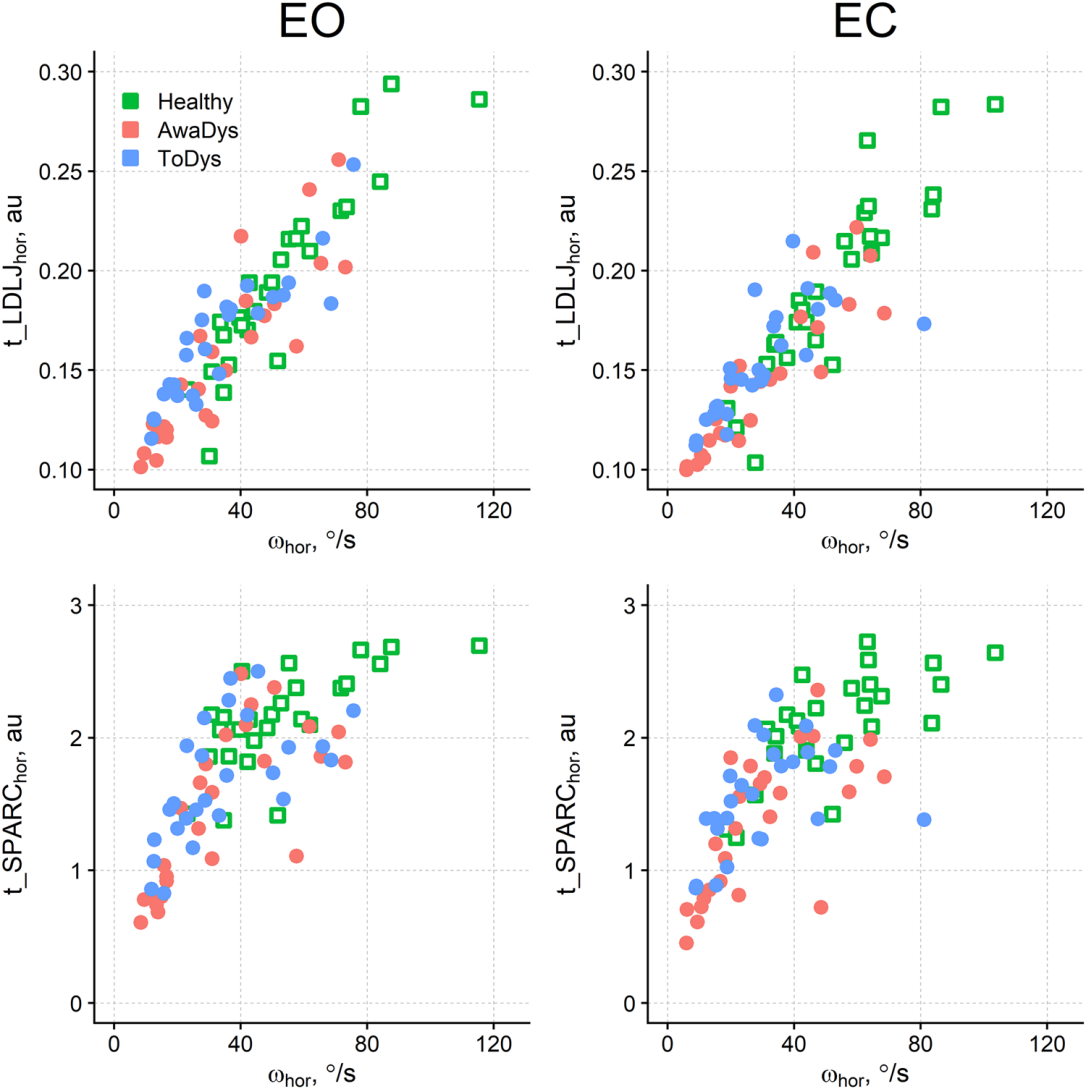
Correlation between the velocity of the head rotation and its smoothness. Same abbreviations as Fig. 1.

Figure 2 shows the relationship between movement velocity on the horizontal plane (ω_hor_) and smoothness measures in the two visual conditions, highlighting that slow movements are less smooth than the fast ones. A positive correlation was found between ω_hor_ and t_LDLJ_hor_ for healthy, AwayDys, and ToDys movements, in both visual conditions (Spearman’s *rho* range 0.87–0.94, *P* corrected according to Bonferroni: < 0.001). Even if weaker, a significant, positive correlation was found between ω_hor_ and t_SPARC_hor_ in the six conditions (Spearman’s *rho* range 0.58–0.83, *P* corrected according to Bonferroni: < 0.016).

After correcting for multiple comparisons, no significant correlation was found between rotation amplitude θ_hor_ and t_LDLJ_hor_, and between θ_hor_ and t_SPARC_hor_.

When θ_hor_ and ω_hor_ were included as covariates in the statistical comparisons, both were significant predictors of t_LDLJ_hor_ (*P* < 0.001). Moreover, the differences found among healthy, AwayDys, and ToDys movements (*χ*^2^_2_ = 3.60, *P* = 0.165), and between the two visual conditions (*χ*^2^_1_ = 0.61, *P* = 0.435) disappeared. The interaction between movement type and vision was not significant as well (*χ*^2^_2_ = 0.44, *P* = 0.802).

By contrast, the same analysis conducted on t_SPARC_hor_, confirmed the results found without including θ_hor_ and ω_hor_ as covariates. In particular, a significant difference was found among movement types (*χ*^2^_2_ = 31.04, *P* < 0.001), with t_SPARC_hor_ being smaller in AwayDys (1.48, 95% CI 1.35–1.61) than in ToDys (1.75, 95% CI 1.62–1.89; *P* < 0.001) and being larger in controls’ movement (1.88, 95% CI 1.72–2.03) than in AwayDys (*P* < 0.001). As before, t_SPARC was not significantly different between visual conditions and no interaction between movement type and vision was found.

### Correlation between rotation measures and TWSTRS scale and additional analyses

No correlation was found between movement amplitude, velocity and smoothness measures and the TWSTRS score, both total (*rho* absolute value ≤ 0.26; *P* uncorrected for multiple comparisons ≥ 0.200) and severity (*rho* ≤ 0.15; *P* ≥ 0.479).

As reported at the beginning of the Results section, ten patients had laterocollis and torticollis, and six patients had dystonic tremor. Laterocollis and dystonic tremor did not affect the TWSTRS total and severity scores. t_SPARC_hor_ was also similar in the different groups (i.e. torticollis vs torticollis and laterocollis; with vs without tremor). Complete results of these additional analyses can be found in Supplementary Information 1.

## Discussion

We showed here that head rotation in cervical dystonia is reduced in amplitude (especially toward the dystonic side) and speed with respect to healthy controls. In addition, net of the difference in these two parameters, smoothness of head movements, as measured by t-SPARC_hor_, is also decreased.

On a strict clinical basis, poor smoothness of movement is easily expected in cervical dystonia. Indeed, voluntary movement in dystonia is known to be possibly corrupted by small-amplitude, high-frequency tremor^26,27^ and, in a recent classification, the adjective “tremulous” has been introduced to describe the dystonic movement^28^. It is also known that, along with abnormal posturing, cervical dystonia can result in twisting and repetitive movements of the head^29^.

Intermittency of voluntary head and arm movements (i.e. reduced smoothness) has been previously described in cervical dystonia^30^, task-specific dystonia^31^, and Parkin disease, an early-onset parkinsonism characterised by segmental dystonia^32^. However, to our knowledge, the effects of movement amplitude and velocity on smoothness have never been considered. With this regard, it is noteworthy that a relationship between smoothness and movement amplitude and velocity has been reported for head motion in healthy controls^33^. In line with other Authors, we firmly believe that movement amplitude and velocity should always be considered when new movement measures are studied^24,34,35^ to avoid wrong conclusions (see the t_LDLJ_hor_ results).

A growing body of evidence suggests that the study of movement smoothness is an essential field for both researchers and clinicians. Sound movement is fast (when required), accurate and smooth^36^. Smoothness of movement is one of the invariant characteristics of healthy voluntary movements^22^ that are actually programmed to be as smooth as possible. For example, even a simple point-to-point movement (like the one here analysed) can be completed with unlimited combinations of trajectories and velocities. Mathematical modelling has shown that the nervous system can choose, among all these possible combinations, the one associated with the minimum jerk^22^ to produce the smoothest possible movement^37^.

It has been rightly pointed out that therapeutic exercise/rehabilitation (i.e. one of the main therapies for the diseased movement, to date) should be targeted in the first place to reduce the motor impairment, thus promoting a true motor recovery. Motor compensation is the next solution if the motor impairment is not amendable^38^. Cervical dystonia is a disease of the neck. Thus, rehabilitation treatments promoting genuine motor recovery should improve head movements by improving neck functioning. In the specific case analysed here, this means to increase the head speed (or the amplitude of the head movement) with respect to the thoracic outlet, rather than increasing head speed (or amplitude) using a trunk rotation (i.e. with motor compensation).

This scenario requires a thorough knowledge of the physiological movement, which should go much further than its kinematic description. Accordingly, given that sound movement is characteristically smooth^22^, therapies aimed at improving movement should also improve smoothness. With this regard, it is noteworthy that smoothness measures have been chosen as outcome measures in randomised clinical trials in different diseases^39–42^. For the clinician, it is also of interest that smoothness measures can differentiate between different levels of impairment^43^ and that poor smoothness is associated with poor outcomes^44^.

From a motor control perspective, the study of movement smoothness is the study of motor coordination^24^, with poor timing and inappropriate intensity of muscular activation (i.e. poor coordination in the motor units’ recruitment) leading to movement jerkiness. The head rotations tested here can be considered a variant of the ballistic (i.e. rapid and discrete) movement. Virtually all rapid movements between two points in space (e.g. wrist flexion) are sustained by the triphasic pattern consisting in the sequential activation of movement’s agonist muscles (i.e. wrist flexors muscles), antagonists (i.e. wrist extensor muscles) and again agonists. More pronounced in very fast movements, the triphasic pattern can be found over a range of movement’s velocities to disappear in very slow movements eventually^45,46^. Apparently, poor coordination within the triphasic pattern can lead to a jerky movement. For example, early activation of the antagonist burst would produce an inappropriate deceleration, i.e. a dip in the velocity profile, thus making the movement jerky. Poor timing of muscular activation, even in simple ballistic movements, is well known in dystonia^47^.

The main result here is that head rotation in the horizontal plane in cervical dystonia is less smooth than in healthy controls, especially in the AwayDys direction, which is also characterised by increased secondary (spurious) movements in the coronal plane. We propose that reduced smoothness in cervical dystonia happens because of the poor coordination between the pathological (i.e. dystonic) muscular contraction and the one produced by the voluntary movement. For example, consider a person with cervical dystonia with pure torticollis causing his/her resting head to be rotated to the left and a healthy control voluntarily resting with his/her head turned to the left. For simplicity, let’s assume that the resting head rotation in both participants is sustained by the tonic contraction of the ipsilateral (left) splenius capitis^48^. When both subjects rotate their head to the right, the healthy participant likely inhibits the contraction of his/her left splenius before activating the right one.

On the contrary, being involuntary, the patient’s dystonic contraction of the left splenius is not (or sparsely) inhibited. This seems a plausible hypothesis, given that muscular co-contraction is a hallmark of dystonia^49^. According to this, two opposite forces act during head rotation in patients with cervical dystonia, with head movement determined by the resultant force from the counteracting voluntary and dystonic contractions. When working against an opposing force, the force produced by the dystonic muscles is highly variable in time^50^, eventually producing dips and peaks (i.e. jerkiness) in the movement’s velocity profile. In this view, smoothness is poor when moving AwayDys because the voluntary contraction deals with the unpredictable antagonistic pathological one.

Smoothness of movement is also poorer when rotating AwayDys than ToDys. This could occur in line with the “tug-of-war” described above because, in ToDys movements, the motor command turns off the voluntary contraction needed to keep the head in the physiological resting position (see “Methods”). Thus the dystonic contraction moves back the head to its (pathological) resting position. In other words, the voluntary motor command lets the dystonic contraction run free.

The current work showed no substantial difference between head rotations with the eyes open and closed, not only in controls but also in patients, a rather unexpected finding. Patients are often asked to move with their eyes open and closed in the neurological examination. This test is also part of the examination of patients with cervical dystonia. Patients often rely on sight to cope with their pathological movement, with the most dramatic example coming from patients with sensory ataxia^51,52^. To note with this regard, reliance on vision seems just the way everyone faces a new and/or challenging movement (e.g. a new piano exercise).

However, the movement tested here is an effortless, ballistic movement. Sensory afferents, vision included^53^, scarcely regulate this type of movement. Another explanation is that the instrumental analysis and clinical examination could assess different movement features. For example, the neurological examination could be focussed more on the fixed postures caused by the disease, which were not considered here, rather than on voluntary movement (see also below).

In the current work, we used two different measures (t_LDLJ and t_SPARC) to quantify movement smoothness. Although these are considered among the most common smoothness measures, many other indices have been proposed^9^. In some respects, this abundance of smoothness measures is not ideal. For example, based on the smoothness measure chosen, different conclusions can be reached about smoothness recovery^24^, clearly a non-optimal finding. However, it should be stressed that some smoothness indices actually quantify variability rather than smoothness^54^.

We showed here that, once movement amplitude and velocity are considered, the transformed LDLJ and SPARC return different results, given that the former does not distinguish between healthy and cervical dystonia, while the latter does. However, this finding should not be considered a contradiction and can have different explanations. Given that the mathematics behind these measures is quite different, LDLJ and SPARC likely quantify different aspects of smoothness^23^. Compared to t_LDLJ, t_SPARC seems more sensitive to changes in smaller movements^55^. In addition, t_SPARC is more robust to measurement noise^9^ being, therefore, more reliable. It is worth mentioning that discrepancies between SPARC and LDLJ’s findings have already been reported^56^.

There was no relationship between any of the four movement measures and the TWSTRS severity and total scores. This, however, does not affect the validity of the current findings. The lack of correlation between smoothness and TWSTRS is not surprising, given that movement jerkiness (and, more generally, movement’s quality) is neglected in this scale. Also, the absence of correlation between TWSTRS and movement amplitude and velocity can be easily explained. First, scales for cervical dystonia do not rate movement’s speed^6^. With this regard, in agreement with our results, it has been shown that the velocity of head rotations did not correlate with the TWSRS total score, while that of flexion–extension and lateral bending movements did^6^. Second, scales assessing the severity of cervical dystonia (TWSTRS included) are significantly focused on the amplitude of head deviation at rest^57^, rather than on the impairment of voluntary movements^58^.

Among the limitations of the current work, we acknowledge that just a single, simple movement has been investigated. Future investigations should verify that these findings also hold for more complex movements, such as cyclic head rotations in the first place. The study of the electromyographic activation of neck muscles also seems of interest. For example, electromyography could further evaluate the hypothesis that smoothness is lower in AwayDys than ToDys movements because of the impaired relaxation of the dystonic muscles in the former condition (see above).

Another intriguing investigation consists in the smoothness analysis when augmented feedback of movement is provided^59^, which (on a clinical basis) is expected to reduce jerkiness.

Ancillary analyses also showed that smoothness of movement is comparable in patients with and without dystonic tremor (see Supplementary Information 1). However, this should be considered a provisional finding due to the small number of patients with dystonic tremor recruited here.

As reported above, several smoothness and variability measures have been developed, and thus, a comparison between these metrics and LDLJ and SPARC could be of interest. However, it should be stressed that the measures used here are recognised as the most valid measures of movement smoothness^9^.

Finally, treatments, such as the botulinum neurotoxins and neurorehabilitation, have been shown to increase the amplitude and velocity of head movements in cervical dystonia^5^. Therefore, the most natural continuation of the current work likely consists in the study of the smoothness modification after cervical dystonia’s treatments, botulinum neurotoxins and rehabilitation exercise included^59^.

## Methods

We conducted an observational, cross-sectional study in which 26 patients with cervical dystonia and 26 healthy controls were consecutively recruited from March 2016 to June 2020.

The current work is part of a larger study aimed to evaluate the therapeutic efficacy of the association of exercise and neurobotulinum toxin in cervical dystonia.

The study was approved by the ethical committee of the IRCCS Don Carlo Gnocchi Foundation (section of the Comitato Etico IRCCS Regione Lombardia) and recorded at ClinicalTrials.gov (NCT03247868). Each participant gave his/her written informed consent to participate in the study. All methods were carried out in accordance with relevant guidelines and regulations, and the study complies with the Declaration of Helsinki.

### Participants

Patients were included following these inclusion criteria: (i) diagnosis of idiopathic cervical dystonia according to Albanese et al.^28^; (ii) age between 18 and 80 years, and (iii) disease duration ≥ 1 year. Patients’ exclusions criteria were: (i) neck skeletal abnormalities (e.g. cervical stenosis); (ii) any clinical sign of cervical myelopathy or cervical radiculopathy; (iii) an additional neurological disorder (e.g. stroke); (iv) therapy with botulinum neurotoxins less than three months before the study enrolment.

Healthy controls were recruited according to a single inclusion criterion, i.e., between 18 and 80 years. Controls’ exclusions criteria were: (i) history of chronic neck pain or low back pain; (ii) history of acute neck pain or low back pain in the three months preceding the experimental session; (iii) the use of pain killer because of neck pain, low back pain or headache in the week before the experimental session; (iv) fear of movement (neck or back); (v) any disease causing activity limitation or participation restriction.

Patients were recruited from the botulinum neurotoxins outpatient clinic of the IRCCS Fondazione Don Carlo Gnocchi, Milan (Italy). Controls were recruited from visitors and personnel of the same facility.

Cervical dystonia is commonly classified by the anatomical plane where the deviation of the head from the physiological resting position is maximal. Accordingly, three main phenotypes of cervical dystonia are recognised. At rest, the head of patients with torticollis is abnormally rotated in the horizontal (hor) plane. In contrast, in laterocollis the head is deviated in the coronal (cor) plane and in antero-retrocollis it deviates in the sagittal (sag) plane.

The current work recruited patients affected by torticollis, with or without some degree of antero-retrocollis and laterocollis. Patients with pure antero-retrocollis or laterocollis or head shift (i.e. an anterior–posterior or left–right translation of the head in the horizontal plane) were excluded.

Patients with Fahn’s tremor (i.e. tremor *regardless of* dystonic posturing) were also excluded, while patients with dystonic tremor (i.e. tremor *in addition to* dystonic posturing) were included^60^.

### Experimental session

At the beginning of the experimental session, the TWSTRS^61^ was collected for each patient. The TWSTRS total score is likely the most common outcome measure in therapeutic trials in cervical dystonia^62^. It consists of three domains: severity, disability and pain. Severity is a scale administered by a clinician, while disability and pain are questionnaires completed by patients. The severity domain measures the impairment’s severity. The disability one collects the self-rated difficulties in completing activities (e.g. reading, watching television). Finally, the pain domain investigates pain duration and severity. High scores indicate a severe disease, increased disability and great pain.

Head movements of the participants were measured using a 9-camera optoelectronic system (SMART-D, BTS, Milan, Italy) able to record the 3D coordinates of passive markers positioned on the body at a sampling frequency of 200 Hz.

**Figure 3:**
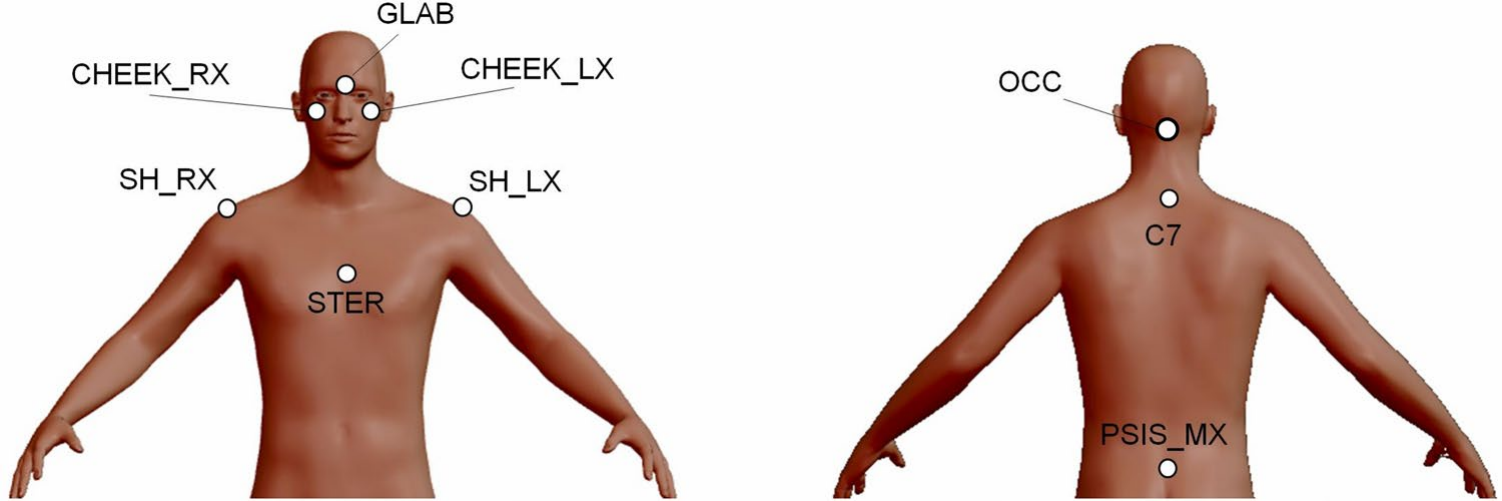
Markers applied to participants. The labels of all markers are indicated. *GLAB* glabella, *CEEK_RX* right cheekbone, *CEEK_LX* left cheekbone, *SH_RX* right acromion, *SH_LX* left acromion, *STER* xiphoid process, *OCC* base of the occipital bone, *C7* seventh cervical vertebra, *PSIS_MX* midpoint between the two posterior superior iliac spines.

Participants were equipped with nine markers applied on the bony landmarks reported in Fig. 3, and were asked to rotate their head to the right and the left, with both their eyes open and closed. Each of the four movements was repeated three times.

Participants sat on a chair and were asked to lean against the seatback and look straight ahead, maintaining the head in its physiological resting position. For patients, this meant countering to some extent the dystonic resting position.

Participants were instructed to move in accordance with the following command: *“turn your head as you should look at something to your far right or far left, hold the position for a few seconds and then come back to the resting position”.* Before recording, the experimenter showed the movement to the participant, who was asked to repeat it a few times.

When ready, participants started the movement after a “go” signal verbally given by the experimenter. In line with previous studies^6^, participants were asked to complete the movement at their comfortable speed.

### Data processing

Markers’ coordinates were low-pass filtered at 6 Hz (5th order, zero-lag, Butterworth filter)^63^ and the time course of head’s angles, relative to the trunk, in the horizontal, coronal, and sagittal planes were computed following published methods^64^ (see Supplementary Information 2 for more details). The head’s angular velocities in the three planes were calculated as the first time derivative of the respective angle.

The current analysis was focused on the rest-to-right and rest-to-left head movements, while the steady phase and the return movements have not been analysed. Onset and termination of movement were defined as the first frames at which the modulus of the angular velocity exceeded and fell below the 5% of its maximum value, respectively.

The following variables were then computed from each movement of the head.

- θ (°): amplitude of head rotation movements;
- ω (°/s): mean angular velocity;
- LDLJ (a.u.): logarithm of the time integral of the squared jerk (third time derivative of the angular displacement), normalised to the amount of head rotation and the duration of movement, as previously detailed^65^;
- SPARC (a.u.): negative arc length (length along a curve) of the Fourier magnitude spectrum of the angular velocity profile (see Balasubramanian et al.^9^ for more details).

θ and ω were computed for all planes, while smoothness measures (LDLJ and SPARC) were calculated for the rotation in the horizontal plane only, since it was the main movement based on the instructions to participants.

To obtain real smoothness (rather than jerkiness) indices, LDLJ and SPARC were transformed into t_LDLJ = 1/LDLJ and t_SPARC = 1/log(− SPARC), respectively. With this transformation, higher values of both measures indicate higher movement smoothness. Moreover, thanks to this transformation, the assumptions of the statistical tests run here are complied in full (see below).

MATLAB (The Mathworks, MA) was used for data processing.

### Statistical analysis

In patients, head movements were termed “away from dystonia” (AwayDys) and “towards dystonia” (ToDys) depending on whether the head rotation improved or worsened the resting head deviation (i.e. the dystonic posture), respectively. For example, if torticollis caused the head to be rotated at rest to the patient’s right, AwayDys (i.e. the movement away from dystonia) was from the rest position to left, while ToDys (i.e. the movement towards dystonia) was from rest to the right. Measures from movements of the same type (e.g. the three head rotations in the AwayDys direction completed with open eyes) were averaged for each participant.

In healthy controls, measures of head rotations towards the dominant and non-dominant sides were aggregated since no significant differences were found between them (Supplementary Information 3). Control’s movements were then referred to as “healthy movement”, independently from their direction (i.e., rightward or leftward).

For each measure (i.e. θ, ω, LDLJ and SPARC), generalised linear mixed-effects models^66^ followed by the Type III Wald chi-square (χ^2^_degrees of freedom_) tests were used to evaluate the effect of movement type (healthy, AwayDys, ToDys), vision (open versus closed eyes), and that of the interaction between movement type and vision. In addition, the same analyses were used to verify if any difference in movement smoothness between controls and patients was due only to a between-groups difference in movement amplitude or speed, or was an independent feature of cervical dystonia. z-tests calculated from the model’s least-squares means, and the corresponding standard errors were used as post-hoc tests.

Spearman correlation was used to evaluate the association between movement measures and the TWSTRS score (total and severity).

The significance level was set at 0.05, and the Bonferroni correction was applied to the post-hoc tests and the correlation analysis because of multiple testing.

Statistical analysis was run in R^67^.

## Supplementary Information

Supplementary Information.

## Data Availability

Data are available from the corresponding author upon reasonable request.
