# Supplementary material for: Smoothness of movement in idiopathic cervical dystonia
Source: Sci Rep. 2022 Mar 24;12:5090. doi: 10.1038/s41598-022-09149-1 (PMC8948176; doi:10.1038/s41598-022-09149-1)
Supplement: Supplementary file 1 — Supplementary Information. [file 41598_2022_9149_MOESM1_ESM.docx]

**SUPPLEMENTARY MATERIALS 1**

**Comparison between patients with torticollis and torticollis plus laterocollis**

The study recruited 26 patients affected by cervical dystonia with torticollis as the main feature. However, ten patients had laterocollis and torticollis, and six patients had dystonic tremor. Only one patient had laterocollis and dystonic tremor. The principal analysis reported in the main text was run on the entire sample of 26 patients. In this additional analysis, the different groups of patients are contrasted (i.e. torticollis vs torticollis plus laterocollis; with vs without dystonic tremor).

The TWSTRS severity score was comparable in patients with torticollis (median = 15.5, interquartile range, IQR = 4.8) and patients with torticollis plus laterocollis (median = 19.0, IQR = 2.0; Wilcoxon rank sum test: P = 0.161). The TWSTRS total score was also similar in the two patients’ groups (torticollis: median = 36.1, IQR = 10.7; torticollis and laterocollis: median = 34.0, IQR = 9.8; Wilcoxon rank sum test: P = 0.979).

The median TWSTRS severity score was the same in patients with (median = 17.0, interquartile range, IQR = 4.3) and patients without dystonic tremor (median = 17.0, IQR = 5.8; Wilcoxon rank sum test: P = 0.501), as well as the TWSTRS total score (with tremor: median = 39.0, IQR = 9.8; without tremor: median = 34.3, IQR = 8.9; Wilcoxon rank sum test: P = 0.235).

Regarding the prime movement (i.e. the movement in the horizontal plane), t_SPARC_hor_ was comparable in the different groups of patients (Supplementary Table 1). θ_hor_ was larger in patients with tremor compared with patients without tremor. ω_hor_ was slightly larger in patients with tremor and patients with laterocollis.

|  |  | **Chisq** | **Df** | **P-value** | **1° group**  *Mean (95% CI)* | **2° group**  *Mean (95% CI)* |
| --- | --- | --- | --- | --- | --- | --- |
| **θ** | **TC + LC vs TC** | 0.89 | 1 | 0.346 | 61.2 (52.0 - 70.4) | 57.2 (50.6 - 63.8) |
|  | **with vs without DT** | 7.17 | 1 | 0.007 * | 65.9 (55.1 - 76.6) | 52.5 (46.9 - 58.1) |
| **ω** | **TC + LC vs TC** | 4.48 | 1 | 0.034 * | 44.2 (31.3 - 57.2) | 31.4 (22.0 - 40.7) |
|  | **with vs without DT** | 5.25 | 1 | 0.022 * | 45.8 (30.7 - 61.0) | 29.7 (21.9 - 37.6) |
| **t_SPARC** | **TC + LC vs TC** | 1.39 | 1 | 0.238 | 1.70 (1.31 - 2.08) | 1.48 (1.21 - 1.76) |
|  | **with vs without DT** | 0.83 | 1 | 0.362 | 1.69 (1.23 - 2.14) | 1.49 (1.26 - 1.73) |

**Supplementary Table 1.** **Head rotation in the horizontal plane in patients with torticollis plus laterocollis and with dystonic tremor.** θ: amplitude of head rotation; ω: mean angular velocity of the head; t_SPARC: transformed Spectral Arc length, 1/log(-SPARC). hor: horizontal plane; TC: torticollis; LC: laterocollis; DT: dystonic tremor; Chisq: chi-square; Df: degrees of freedom; 95% CI: 95% confidence interval. 1° group: TC + LC or with DT; 2° group: TC or without DT. Type III Wald chi-square tests were calculated on regression models (see main text and Supplementary materials 2). The following categorical variables were used as predictors: group (TC vs TC + LC) and tremor (with vs without). θ, ω and t_SPARC were thus averaged across the "eyes" and "movement direction" conditions.

**SUPPLEMENTARY MATERIALS 2**

**Calculation of head angles**

The angles of the head relative to the trunk were computed following the notion that the availability of the 3D coordinates of three markers on each body segment allows for the identification of its local reference frames (1,2).

In particular, based on Figure 1 of the main text, the trunk reference frame was defined by an upwards longitudinal Y-axis passing through PSIS_MX and the midpoint between C7 and STER, a forward-oriented X-axis perpendicular to the plane identified by the trunk longitudinal Y-axis and by the SH_RX-SH_LX vector, and a Z-axis computed as the cross-product of X and Y-axis. The head reference frame was defined by a right-oriented Z-axis passing though CHEEK_LX and CHEEK_RX, an upwards Y-axis perpendicular to the plane identified by the head Z-axis and by the vector passing through the OCC and the midpoint between CHEEK_RX and CHEEK_LX, and a forward-oriented X-axis computed as the cross-product between Y and Z axis.

As previously described (1,2), head angle (relative to the trunk) in the sagittal, coronal, and horizontal planes were computed as the angles of the head local frame with respect to the trunk local reference frame.

**Details on the generalised linear mixed-effects models.**

Mixed-effects models were used for the principal analysis. Generalised linear mixed-effects models (GL-MEMs) with gamma distribution were chosen in most cases. The choice fell on the GL-MEMs since the assumptions of linear models (i.e. normality and constant variance of residuals (3)) were not met, and the frequency distribution of the response variables was skewed.

In the analysis on healthy controls, sight (i.e. eyes open, EO vs eyes closed, EC), movement direction (i.e. to-dominant, ToDom vs to-non-dominant, ToNoDom) and their interaction were specified as fixed effects. Sight, movement type, i.e. controls' movement (healthy), patients' away dystonia (AwaDys) and patients' towards dystonia (ToDys) movements, and their interaction were the fixed effects of the analysis run on controls and patients.

Regarding random effects, models with random intercepts and slopes were tested. In each model, participants were set as random intercepts and both predictors (i.e. sight, movement direction, movement type), but not their interaction, were specified as random slopes (maximum complexity model (3)).

Analysis-of-variance tables were calculated from MEMs. Type III Wald chi-square tests were used to assess if the models' explanatory variables were significant predictors of the response variable. With this approach, the output of the MEMs calculated here can be read similarly to the output of the traditional ANOVA (repeated measures or mixed model) and ANCOVA (mixed model).

According to Bonferroni, the significance level of the post-hoc comparisons was adjusted for multiple testing.

**REFERENCES**

1. Rabuffetti M, Marzegan A, Crippa A, Carpinella I, Lencioni T, Castagna A, et al. The LAMB gait analysis protocol: Definition and experimental assessment of operator-related variability. Proc Inst Mech Eng H. marzo 2019;233(3):342–53.

2. Grood ES, Suntay WJ. A joint coordinate system for the clinical description of three-dimensional motions: application to the knee. J Biomech Eng. maggio 1983;105(2):136–44.

3. Faraway JJ. Extending the Linear Model with R: Generalised Linear, Mixed Effects and Nonparametric Regression Models, Second Edition. 2° edizione. Boca Raton: Chapman and Hall/CRC; 2016. 413 pag.

**SUPPLEMENTARY MATERIALS 3**

**Head movements in healthy controls**

As shown in Supplementary Table 1, the amplitude of head rotation (θ) towards the dominant (ToDom) side was similar to the one towards the non-dominant (ToNoDom) side in the horizontal and sagittal planes. A difference between ToDom and ToNoDom movements was found only for θ in the coronal plane. However, this difference was quite negligible (about 1.5 °).

For all the three rotation planes, the mean angular velocity of the head was similar between ToDom and ToNoDom movements.

Smoothness measures (t_LDLJ and t_SPARC), computed on the main horizontal rotation only (see Methods in the main document), were also comparable between movement sides.

| **Rotation Plane** | **Variable** | **Movement Direction** | | **Statistics** | |
| --- | --- | --- | --- | --- | --- |
|  |  | ToDom  *Mean (95% CI)* | ToNoDom  *Mean (95% CI)* | Wald χ^2^_1_ | p-value |
| Horizontal | θ (°) | 62.8 (58.8 - 66.7) | 64.4 (60.8 - 68.0) | 0.97 | 0.326 |
|  | ω (°/s) | 51.7 (41.6 - 61.8) | 54.2 (44.4 - 64.0) | 1.37 | 0.242 |
|  | t_LDLJ (a.u.) | 0.214 (0.18 - 0.24) | 0.215 (0.18 - 0.24) | 0.34 | 0.559 |
|  | t_SPARC (a.u.) | 2.22 (2.01 - 2.43) | 2.24 (2.02 - 2.47) | 0.32 | 0.571 |
| Coronal | θ (°) | 17.1 (14.1 - 20.0) | 18.7 (15.9 - 21.6) | 4.30 | 0.038 |
|  | ω (°/s) | 16.6 (12.6 - 20.5) | 18.0 (14.1 - 21.8) | 2.57 | 0.109 |
| Sagittal | θ (°) | 10.6 (7.8 - 13.4) | 9.6 (7.2 - 12.1) | 3.40 | 0.065 |
|  | ω (°/s) | 7.6 (5.4 - 9.9) | 6.7 (4.2 - 9.3) | 2.74 | 0.100 |

**Supplementary Table 2.** **Parameters describing head rotation in healthy controls.** 95% CI: 95% Confidence Interval; ToDom: head rotation towards the dominant side; ToNoDom: head rotation towards the non-dominant side; θ: amplitude of head rotation; ω: mean angular velocity of the head; t_LDLJ: transformed log dimension-less jerk (1/LDLJ); t_SPARC: transformed Spectral Arc length [1/log(-SPARC)]. P-values indicate the comparison results between ToDom and ToNoDom movements, Wald chi-square (χ^2^) test.
